# Frequency dependence of cortical somatosensory evoked response to peripheral nerve stimulation with controlled afferent excitation

**DOI:** 10.1088/1741-2552/adc204

**Published:** 2025-03-28

**Authors:** Disha Gupta, Jodi Brangaccio, Helia Mojtabavi, Jonathan S Carp, Jonathan R Wolpaw, N Jeremy Hill

**Affiliations:** 1 US. Department of Veterans Affairs, National Center for Adaptive Neurotechnologies, Stratton VA Medical Center, Albany, NY, United States of America; 2 Electrical and Computer Engineering Department, State University of Albany, Albany, NY, United States of America; 3 School of Public Health, State University of New York at Albany, Albany, NY, United States of America

**Keywords:** electroencephalography, tibial nerve stimulation, Hoffman reflex, somatosensory evoked potential, operant conditioning, neurorehabilitation

## Abstract

*Objective.* H-reflex targeted neuroplasticity (HrTNP) protocols comprise a promising rehabilitation approach to improve motor function after brain or spinal injury. In this operant conditioning protocol, concurrent measurement of cortical responses, such as somatosensory evoked potentials (SEPs), would be useful for examining supraspinal involvement and neuroplasticity mechanisms. To date, this potential has not been exploited. However, the stimulation parameters used in the HrTNP protocol deviate from the classically recommended settings for SEP measurements. Most notably, it demands a much longer pulse width, higher stimulation intensity, and lower frequency than traditional SEP settings. In this paper, we report SEP measurements performed within the HrTNP stimulation parameter constraints, specifically characterizing the effect of stimulation frequency. *Approach.* SEPs were acquired for tibial nerve stimulation at three stimulation frequencies (0.2, 1, and 2 Hz) in 13 subjects while maintaining the afferent volley by controlling the direct soleus muscle response via the Evoked Potential Operant Conditioning System. The amplitude and latency of the short-latency P40 and mid-latency N70 SEP components were measured at the central scalp region using non-invasive electroencephalography. *Main* *results.* As frequency rose from 0.2 Hz, P40 amplitude and latency did not change. In contrast, N70 amplitude decreased significantly (39% decrease at 1 Hz, and 57% decrease at 2 Hz), presumably due to gating effects. N70 latency was not affected. Across all three frequencies, N70 amplitude increased significantly with stimulation intensity and correlated with M-wave amplitude. *Significance*. We assess SEPs within an HrTNP protocol, focusing on P40 and N70, elicited with controlled afferent excitation at three stimulation frequencies. HrTNP conditioning protocols show promise for enhancing motor function after brain and spinal injuries. While SEPs offer valuable insights into supraspinal involvement, the stimulation parameters in HrTNP often differ from standard SEP measurement protocols. We address these deviations and provide recommendations for effectively integrating SEP assessments into HrTNP studies.

## 1. Introduction

Over the past few decades, H-reflex targeted neuroplasticity (HrTNP) protocols have shown promise for rehabilitation after brain or spinal injuries [1–6]. The HrTNP protocol is an operant conditioning protocol [7] that can gradually increase or decrease the size of the spinal stretch reflex, or its electrical analog, the Hoffman reflex (H-reflex), by rewarding changes in the appropriate direction (shown in animals [8–12] and humans [2, 3, 5]). This changes the reflex pathway, thereby affecting behaviors such as locomotion, which uses the pathway. Thus, an appropriate HrTNP protocol can improve locomotion in animals and people with spinal cord injury [4, 5, 11] or other neurological disorders such as stroke and multiple sclerosis [8, 13]. This body of work has also added to our mechanistic understanding of plasticity in the central nervous system, also discussed in [14].

Typically, in an HrTNP protocol [1, 2], electrical stimulation of a mixed (sensory and motor) nerve generates a spinal reflex (H-reflex) [15], reflecting the largely monosynaptic activation of spinal motoneurons by group Ia afferent fibers. Simultaneously, the electrical stimulus depolarizes efferent axons to elicit a compound muscle action potential (M-wave) in the muscle innervated by the stimulated nerve. At higher intensities, H-reflex reaches a maximum (referred to as *H*_max_), beyond which the antidromic current starts colliding with the H-reflex and cancelling it, resulting in a bell-shaped curve; the M-wave keeps increasing till it reaches an asymptote (referred to as the *M*_max_) when all muscle spindles are firing synchronously [15]. The *H*_max_ and *M*_max_ are determined via recruitment curve measurements for each individual. Recruitment curve data are then used to find the stimulation intensity that evokes an H-reflex at ∼75% of the *H*_max_ and an M-wave at 10%–20% of the *M*_max_. In the subsequent assessment runs this stimulation intensity is used as the base stimulation intensity, and the M-wave as the target M-wave. Here, the M-wave is monitored and maintained within a predefined range of 10%–20% of the target M-wave, by slightly varying the stimulation intensity as needed. This approach maintains a stable effective afferent excitation and, consequently, stable motoneuron excitation during assessment [16, 17].

As the afferent volley from peripheral stimulation also travels to the cerebral cortex, there is a growing interest in studying the supraspinal responses generated in the cerebral cortex during these paradigms. Cortical somatosensory responses can be measured at the scalp via electroencephalography (EEG) as somatosensory evoked potentials (SEPs) [18–20]. SEPs are well-documented event-related potentials that reflect somatosensory transmission in the dorsal column-lemniscus pathway [20]. Assessing these cortical afferent responses can be useful for investigating somatosensory cortical neuroplastic changes during HrTNP conditioning studies.

However, although SEP and H-reflex measurement procedures have been well-studied individually, with methodological recommendations and normative data available for each [1, 15, 21–24], their combined assessment necessitates careful consideration of parameter settings when comparing studies or when performing longitudinal studies. For example, the recommended stimulation settings for SEPs include monophasic pulses with a duration of 0.1–0.2 ms [21], a stimulation frequency of 2–5 Hz [21, 25], a stimulation intensity of three times the sensory threshold or just above motor threshold (indicated by a small visible twitch in the muscle supplied by the nerve) [21, 26], recording of 200–500 trials [21, 25], and stimulation with the limb at rest. For the HrTNP protocol, the recommended settings are biphasic/monophasic pulses of longer (1 ms) width, a slower stimulation frequency (0.2 Hz), a higher stimulation intensity (higher than twitch threshold, approximately 10%–20% of the maximum M-wave) [1, 2, 4]), a smaller number of trials (20–75), and recording while maintaining low-level muscle contraction in the stimulated limb. While a few studies, such as Kato *et al* [27], have measured SEPs under alternate stimulation parameters, a more detailed evaluation of these parameters and their effects on SEPs—under controlled peripheral excitation—would benefit sensory assessment research, particularly in the context of HrTNP studies.

In this study, we describe a protocol that includes SEP measurements in an HrTNP protocol using typical HrTNP parameter settings for tibial nerve stimulation at the popliteal fossa. The H-reflex and M-wave were measured at the soleus leg muscle using EMG. The background soleus EMG was maintained at a predefined low level sufficient for weight-bearing while standing upright. This is generally 5%–10% of the soleus maximum voluntary contraction (MVC), adequate for maintaining balance, counteracting gravity, and keeping the ankle joint stable [28]. SEPs are measured with non-invasive scalp EEG, with a focus on an early thalamocortical SEP response (P40), which remains unchanged in cortical lesions [29], and a mid-latency cortical somatosensory response (N70) [30, 31], which is abolished in cortical lesions [29], both of which are typically measured at the central scalp region for tibial nerve stimulation [30, 32]. We characterized the effect of stimulation frequency on SEPs in HrTNP settings. We also discuss the advantages and disadvantages of HrTNP-related parameter variations from the recommended SEP settings, which may help reduce measurement variability in multisession SEP assessments in HrTNP conditioning studies.

## 2. Methods and analysis

**Participants:** Thirteen healthy people participated in the study (5 men/6 women, age: 45.7 ± 19.7 years, height: 65.8 ± 4.0 inches). Exclusion criteria included a history of neurological disease, use of neuromodulatory medications, open wounds or known skin infections on the scalp, pregnancy, and metal implants such as cardiac pacemakers, cochlear implants, stimulators, or metal rods. All data were obtained with written informed consent, and the protocol was approved by the local institutional IRB at the Stratton VA Medical Center (#1584762). Two participants had large shoulder and leg movements in response to every stimulus, which injected large movement artifacts into their EEG data. These data were not included in the SEP analysis.

**Experimental Setup:** The experimental protocol was designed to assess the effect of tibial nerve stimulation frequency on cortical SEPs while generating an H-reflex with maintained background EMG and an M-wave size within a narrow range. The data from each participant were collected in a single session. The experimental protocol consisted of two contiguous blocks of six runs each. Each block consisted of a *recruitment run,* followed by a corresponding *assessment run* at each of the three stimulation frequencies (0.2, 1, and 2 Hz). A *Recruitment run* involved generating M-wave and H-reflex recruitment curves to estimate the target stimulation intensity that met the HrTNP criteria for the subsequent assessment run. The HrTNP criterion requires eliciting an H-reflex at approximately 75% of *H*_max_ (i.e. on the ascending edge of its recruitment curve), and an M-wave at 10%–20% of *M*_max_ (referred to as the target M-wave), with a maintained background muscle contraction. The *assessment run* consisted of 75 trials with maintained background soleus muscle contraction and a stable soleus target M-wave. The order of the stimulation frequencies in the first block was randomized across participants, and the second block was conducted in reverse order.

**Peripheral nerve stimulation:** A constant current electrical stimulator (DS8R, Digitimer Ltd) was used to deliver 1 ms biphasic electrical stimulation pulses (positive initial polarity) via surface electrodes placed on the tibial nerve at the popliteal fossa. A 22 × 35 mm electrode (A10041-60, Vermont Medical Inc.) was used as the anode and attached to the apex of the popliteal fossa. A 22 × 22 mm electrode (A10040-60, Vermont Medical Inc.) was used as the cathode and attached 3 cm longitudinally below the anode. These stimulation parameters and electrode placements have been used in previous HrTNP studies [18]. Stimulation was applied at three different intervals that were randomly jittered by 20%, resulting in interstimulus intervals (ISIs) of 5 ± 1 s, 1 ± 0.2 s and 0.5 ± 0.1 s, corresponding to mean frequencies of 0.2 Hz, 1 Hz, and 2 Hz, respectively. A pair of recruitment curves and assessment runs was collected at each of these stimulation frequencies.

*Recruitment Run:* Recruitment curves were obtained for each individual to determine their *H*_max_ and *M*_max_. To generate recruitment curves, the stimulation current amplitude was increased from the H-reflex threshold until the M-wave reached an asymptote at its maximum value (*M*_max_). The stimulation intensity was typically incremented in intervals of 0.1–0.5 mA, in order to reach *M*_max_. The recruitment curves were examined to identify the stimulation intensity that generated an H-reflex at approximately 75% of *H*_max_ and a visible small M-wave, generally approximately 10%–20% of *M*_max_ (referred to as the target M-wave).

*Assessment Run:* Next, 75 trials were acquired at the target stimulation intensity determined from the preceding recruitment run, with small variations to maintain the M-wave within 10%–20% of the Target M-wave. The background soleus EMG was also maintained at a predefined low level (typically equivalent to 5%–10% of the MVC) sufficient for weight-bearing when standing upright. This level of stimulus intensity (with an M-wave of 10%–20% of *M*_max_) induces a soleus muscle twitch, which can cause foot movement. The participants were asked not to actively suppress any foot movements.

**SEP recording:** SEPs were recorded using a 19-channel EEG headset (DSI, Wearable Sensing, CA), with dry active electrodes positioned according to the 10–20 international EEG system [33]. The ground electrode was positioned at FPz, and the reference electrodes at the linked earlobes. The data were acquired using the BCI2000 software [34, 35] at a sampling rate of 300 Hz. For tibial nerve stimulation, the region of interest used was the central scalp area, which is most activated during lower limb stimulation, and is described in the sensory homunculus as the area functionally associated with the lower limbs [36, 37]. The SEP features of interest in this study were the amplitude and latency of the short-latency deflection (P40) and subsequent mid-latency deflection (N70). To synchronize the EEG data with the EMG data, the electrical stimulus pulses sent from the Evoked Potential Operant Conditioning System (EPOCS) to the electrical stimulator were also sent to the EEG data acquisition system via 5 V transistor-to-transistor logic (TTL) pulses.

**Figure 1.**
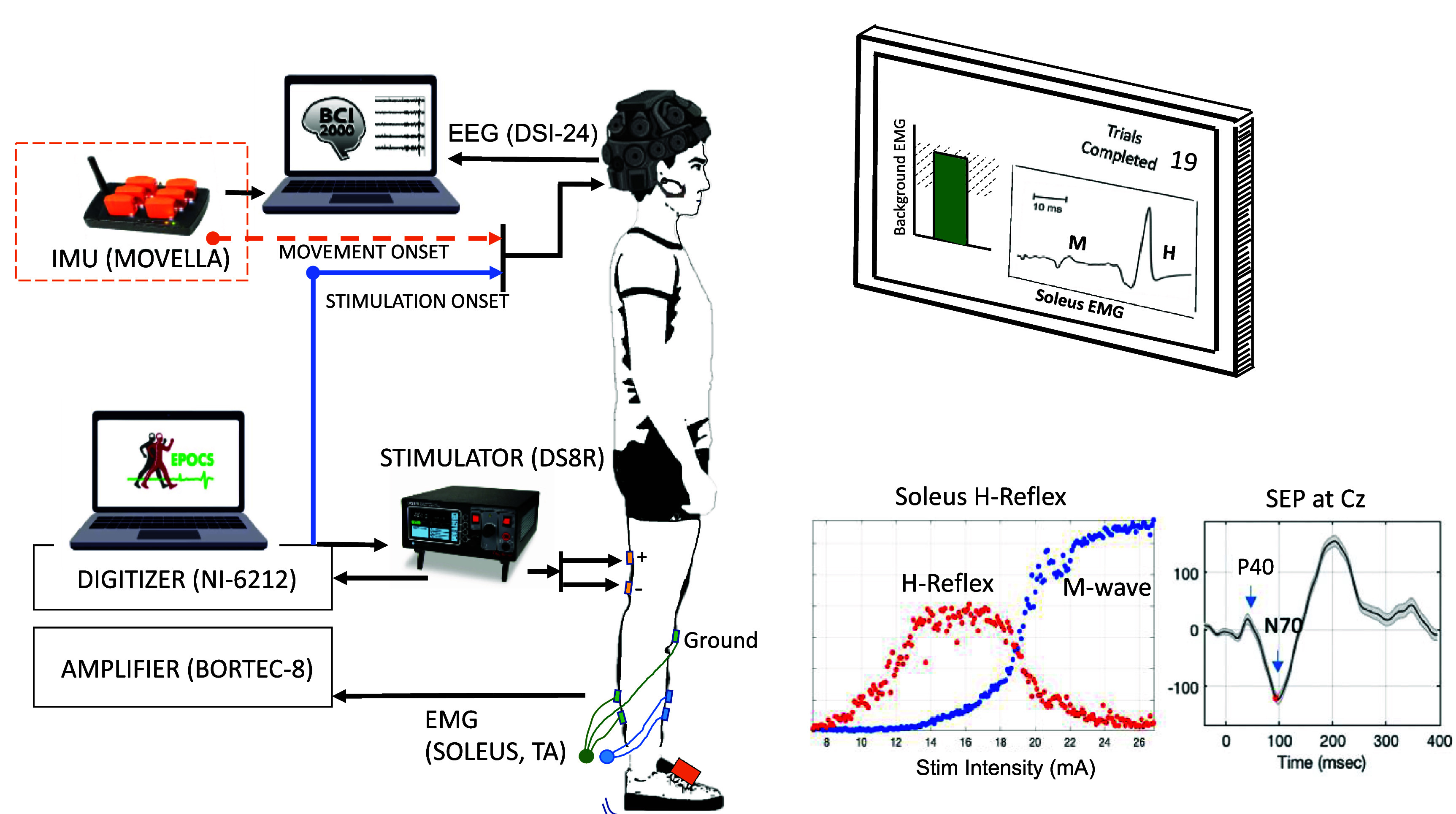
Experimental Setup: the participant stood in a comfortable standing posture, at arm’s length from a wall-mounted monitor. Tibial nerve was stimulated at the popliteal fossa with constant current stimulator DS8R (Digitimer Ltd.). Soleus electromyography (EMG) was recorded with an AMT-8 (Bortec Biomedical Ltd) analog amplifier and a digitizer NI-6212 (National Instruments) using our Evoked Potential Operant Conditioning System (EPOCS [1, 2]). Electroencephalography (EEG) was recorded with DSI-24 (Wearable Sensing) and BCI2000 software [34, 35]. Wireless inertial measurement unit (IMU) sensors (Movella) were used to capture foot movement in three participants. To synchronize the datasets, TTL pulses were transmitted from the EPOCS and IMU hub to the EEG headset. Background EMG was displayed as the height of a vertical bar on the wall monitor, to help the participant maintain EMG within a predefined low range. The M-wave and H reflex was displayed on the screen as a time signal after every stimulation and their recruitment curves were monitored in pseudo-real time. The inset on the right shows the H-reflex and M-wave recruitment curves and the somatosensory evoked potential P40 and N70 response at the Cz electrode, from one of the participants.

**H-reflex and M-wave recordings:** The skin over the soleus and tibialis anterior muscles was cleaned with alcohol swabs and lightly abraded with paper towels. EMG was recorded with bipolar self-adhesive Ag/AgCl surface electrodes (Neuroplus electrodes (22 × 22 mm) (A10040-60, Vermont Medical Inc.) on the soleus muscle of the right leg to record the H-reflex and M-wave, and on its antagonist muscle tibialis anterior to record its ongoing (background) activity. The electrodes were placed longitudinally on the muscle belly, as per the procedures described in Hill *et al* [1], and multiple HrTNP studies [2–4]. The electrode centers were placed 3 cm apart. An additional electrode was placed on the patella for a ground. Signals were recorded with an 8-channel analog signal acquisition system (AMT-8, Bortec Biomedical Ltd, Canada). EMG was pre-amplified with a gain of 500 and bandpass filtered at 10–1000 Hz. The data was digitized with an analog-to-digital convertor (PCIe-6321, National Instruments) at a sampling rate of 3200 Hz. This data was continuously transmitted to a computer running the EPOCS software [1] that displayed the M-wave and H-reflex evoked by each stimulus in real-time (see figure 1). EPOCS continuously monitored soleus and tibialis anterior background EMG. It was setup to send a trigger signal to the stimulator when the average background EMG was maintained within a predefined amplitude range (corresponding to 5%–10% of MVC) for at least 2 s prior to stimulation at 0.2 Hz stimulation frequency, and for at least 200 ms prior to stimulation at 1 or 2 Hz stimulation frequency. Background EMG was continuously displayed to the participant (see figure 1) to help them maintain it within the specified range. Participants maintained a stable standing posture during recording. They were able to sit down between runs, if they desired.

**Inertial measurement unit (IMU) for foot motion capture:** in three participants, we were able to measure twitch-induced foot movement by attaching a wireless IMU (Awinda MTw, Movella, NV), a micro-electro-mechanical embedded system, to the dorsal portion of the feet, as shown in figure 1. MT manager (Movella, NV) software was used to acquire the movements at a sampling rate of 100 Hz, and the data files were exported to the MATLAB format for further processing. At the start and end of the recording, a 5-V TTL pulse was transmitted from the IMU hub to the EEG hardware via a BNC connector, allowing the IMU and EEG datasets to be synchronized.

## 3. Data analysis

*EEG analysis:* EEG data were pre-processed to remove bad channels and filtered at 0.2–40 Hz using a zero-phase causal digital filter (Butterworth, model order 2). Ocular artifacts were identified and removed using independent component analysis [38]. Data were segmented in epochs from −50 to 400 ms relative to the stimulus onset. A Laplacian filter was used for spatial filtering. Single-trial baseline correction was performed by subtracting the mean of the pre-stimulus 50 ms, followed by the removal of bad trials based on the trial statistics. The SEP elicited by tibial nerve stimulation was measured from the channel Cz (central scalp position) as per the somatosensory homunculus for the lower extremities and previous studies showing its activation with lower limb stimulation [39, 40]. A typical SEP elicited by this protocol is shown in figure 1, similar to that described in previous studies [27]. We mainly focused on the peak and latency of the mid-latency positive peak P40 and the subsequent negative deflection N70 at each of the three stimulation frequencies (0.2 Hz, 1 Hz, and 2 Hz). The coefficient of determination (*r*^2^) was determined at Cz with respect to the baseline epochs to assess the signal-to-noise ratio (SNR) of these peaks across trials.

Participant-specific P40 and N70 latencies were identified using the *r*^2^ estimated from 15 ms wide windows, in epochs from 0 to 400 ms, with respect to baseline epochs of 50 ms. The minimum signed *r*^2^ peak within a predefined window of 20–60 ms and 60–165 ms was used to objectively determine the latency of P40 and N70, respectively. Long time windows were used to pre-emptively accommodate any latency shifts across participants and stimulation frequencies. Next, the P40 and N70 peak amplitudes were measured at these latencies and averaged across the runs for each stimulation frequency. For supplementary analysis, P200 was extracted in a similar manner to the P40 and N70, using a predefined window of 150–250 ms.

*EMG analysis:* Epochs from −200 to 400 ms (relative to stimulation onset, defined as 0 ms) were extracted from the continuously recorded data. The EMG was already band-pass filtered (10−1000 Hz) by the amplifier, which adequately removed the low-frequency movement artifacts. For each epoch, H-reflexes and M-waves were quantified by calculating the RMS value of the signal that fell within the investigator-determined time windows, which were the same for all three frequencies for a given participant. For recruitment curve trials, responses were averaged across groups of up to four trials, while for frequency-assessment trials, M-wave and H-reflex responses were averaged across all 75 trials.

To examine the effect of stimulus intensity on the SEP, and to assess the association between SEP and M-waves and H-reflexes, a normalized stimulus intensity was used, normalized by the maximal M-wave (*M*_max_) per recruitment curve per participant [16]. SEP averages were obtained at discreet normalized stimulus intensities based on the percentage of M-wave they elicited—S(M10) for 0%–10% *M*_max_, S(M20) for 10%–20% *M*_max_,…S(M90) for 80%–90% *M*_max_. Alpha motoneuron activation was also estimated at these stimulation intensities by calculating the *H*–*M*_max_ ratio.

*IMU analysis*: The IMU data were used to measure foot movement velocity and displacement due to muscle twitch. The data stream from the sensor on the stimulated leg was exported to MATLAB and synchronized with EEG data based on the trigger pulse recorded in the inertial and EEG datasets. The vertical component of the velocity was calculated from the three-dimensional acceleration data. Data were epoched from −50 to 400 ms, using the stimulation onset triggers, and averaged across trials. The average velocity and displacement across participants were calculated with reference to those at 0.2 Hz.

*Statistical analysis*: All numerical values are presented as the mean ± standard deviation. The SNR of the SEP peaks was analyzed using the coefficient of determination (*r*^2^) between the signal and background EEG. The SNR of the spatial SEP peak distribution was analyzed by averaging the *r*^2^ topographies of each peak across the participants. As most data were not normal (Lilliefors test [41], *p* > 0.05), non-parametric tests were used. The Friedman Repeated measures test was used for the assessment of repeated measures, followed by post hoc tests with Tukey’s multiple comparison procedure. The effect size was estimated using the coefficient of concordance (Kendall’s $w$ [42]) with the equation $w = \,{\chi ^2}/n\left( {k - 1} \right)$, where ${\chi ^2}$ is the Friedman test statistic, $n$ is the sample size, and $k$ is the number of repeated measurements. Correlation analysis was performed with Spearman’s rho; *p*-values less than 0.05 were considered statistically significant. The effect size was estimated using Hedge’s *g* [43, 44] with the equation: $g = \,\left( {\,\overline {\,{x_1}} - \,\overline {{x_2}} \,} \right)/{s_p}$, where $\,\overline {{x_n}} \,$ is the group mean and ${s_p}$ is the pooled standard deviation, calculated as ${s_p} = \,\sqrt {\left( {s_1^2 + \,s_2^2\,} \right)/2} $. The interpretation of both Kendall’s $w$ and Hedge’s *g* was based on Cohen’s interpretation guidelines [43] of 0.1—<0.3 (small effect), 0.3—<0.5 (moderate effect), and ⩾0.5 (large effect). FDR correction for multiple comparisons was used where appropriate [45]. Analysis of covariance was performed using MATLAB 2020b (MathWorks, MA), and post hoc analyses were performed using Tukey’s test for multiple comparisons. All statistical analyses were performed using the MATLAB software.

## 4. Results

SEP P40 and N70 were invariably present and easily recognized at central scalp (Cz) in all participants. SEP was measured while concurrently monitoring the H-reflex and controlling the M-wave of the soleus muscle, using the EPOCS software platform.

**Figure 2.**
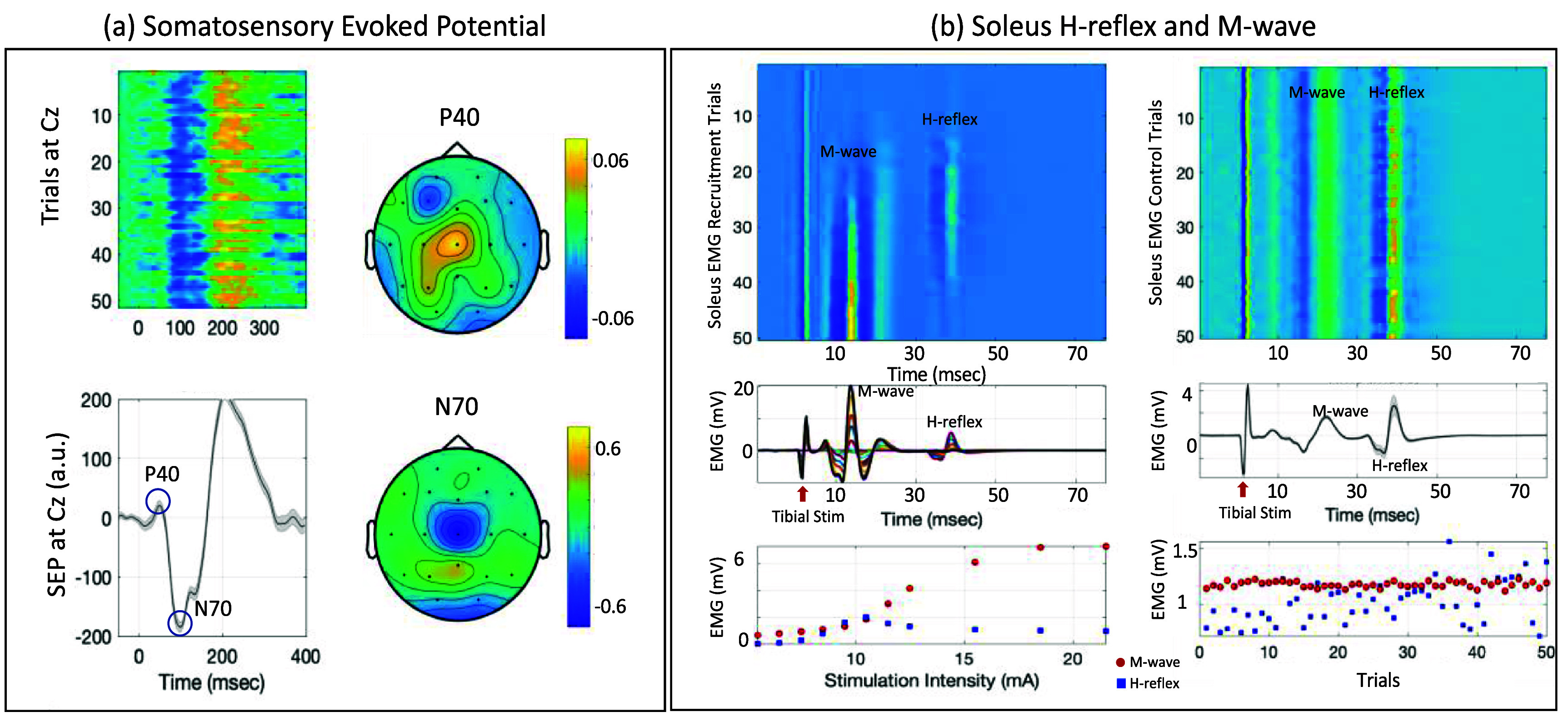
Representative example of (a) somatosensory evoked potential (SEP) and (b) right soleus H-reflex and M-wave, elicited by tibial nerve stimulation at the popliteal fossa. Left panel shows the heatmap of trials from one run, the SEP with P40 and N70 deflections, and the spatial distribution of these peaks via coefficient of determination (*r*^2^). Right panel shows the electromyography (EMG) responses at the soleus muscle for the corresponding recruitment curve and assessment run—trial heatmap, mean EMG signal and the RMS of H-reflex and M-wave responses.

A typical HrTNP protocol is performed at 0.2 Hz, with a 1 ms pulse width, and a stimulation intensity that elicits an H-reflex that is approximately 75% of *H*_max_ (i.e. on the rising edge of its recruitment curve), and an M-wave that is about 10%–20% of *M*_max_. The SEP acquired in this setup is illustrated with a representative example in figure 2. The spatial distributions of P40 and N70 were focused on the central scalp area (Cz), as expected for the lower limb stimulation. The mean latency of P40 and N70 were 42.2 ± 2.1 ms and 96.8 ± 3.1 ms, and the mean amplitudes were 26.7 ± 5.1 a.u. and −141.5 ± 20.8 a.u. The coefficient of determination (*r*^2^) for P40 and N70 amplitudes were 0.03 ± 0.05 and 0.5 ± 0.29 respectively, which illustrates the SNR across trials; it is higher for N70. The *r*^2^ did not change significantly with smaller trial subsets of 10, 20, 30, 40 or 50, for both P40 and N70 (Friedman test, *p* > 0.05 for both).

The H-reflex and M-wave recruitment curves were obtained before each run to determine the average stimulation intensity specific to the participant and the session that met the HrTNP criteria. This was achieved by varying the stimulation intensity from a level that elicited the H-reflex threshold (6.1 ± 2.2 mA) up to the intensity that reached an asymptote for the M-wave, (i.e. the *M*_max_) (24.5 ± 9.0 mA). Characteristic H-reflex and M-wave recruitment curves were observed across all stimulation frequencies for all participants (as illustrated by a representative dataset in figure 2(b), left panel). The H-reflex recruitment curve has a characteristic bell shape, while the M-wave follow a sigmoidal curve [46]. The M-wave was observed at an average latency of 8.0 ± 2.9 ms, and the H-reflex was observed at an average latency of 34.0 ± 3.8 ms, both of which are within the expected ranges [1, 2, 47].

There was some inter-individual variability in the recruitment curves, which is expected. The *M*_max_ exhibited a coefficient of variation across participants of 0.29 at 0.2 Hz, 0.21 at 1 Hz, and 0.18 at 2 Hz. The *H*_max_/*M*_max_ ratio was 0.4 ± 0.3 s.d. across participants at 0.2 Hz, 0.4 ± 0.2 s.d. at 1 Hz, and 0.4 ± 0.2 at 2 Hz. These are similar to values reported in other studies [48].

For the subsequent assessment runs, the initial stimulation intensity was determined from the recruitment curve, such that the H-reflex was 28.9 ± 17.2% of *M*_max_ (i.e. along the rising edge of the H-reflex recruitment curve), the M-wave was 14.4 ± 12.3% of the *M*_max_, and the background EMG was maintained at 1.4 ± 0.05% of *M*_max_ (figure 2(b), right panel). The stimulation intensity was modified as needed during data collection to maintain the target M-wave amplitude. The average absolute stimulation intensity across participants and runs was 10.5 ± 0.6 mA.

**Figure 3.**
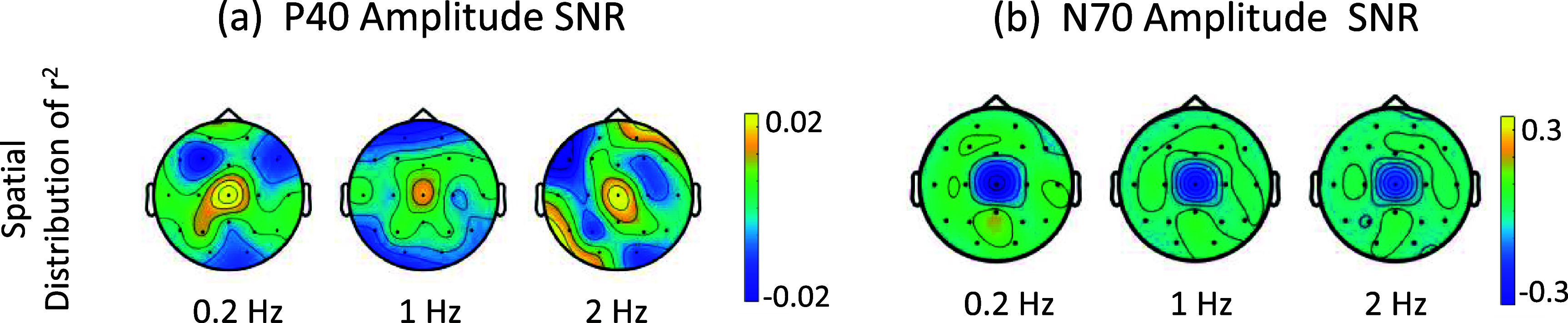
Spatial distribution of the signed coefficient-of-determination (*r*^2^) for (a) P40 and (b) N70 peak amplitudes across participants, at the three stimulation frequencies.

**Figure 4.**
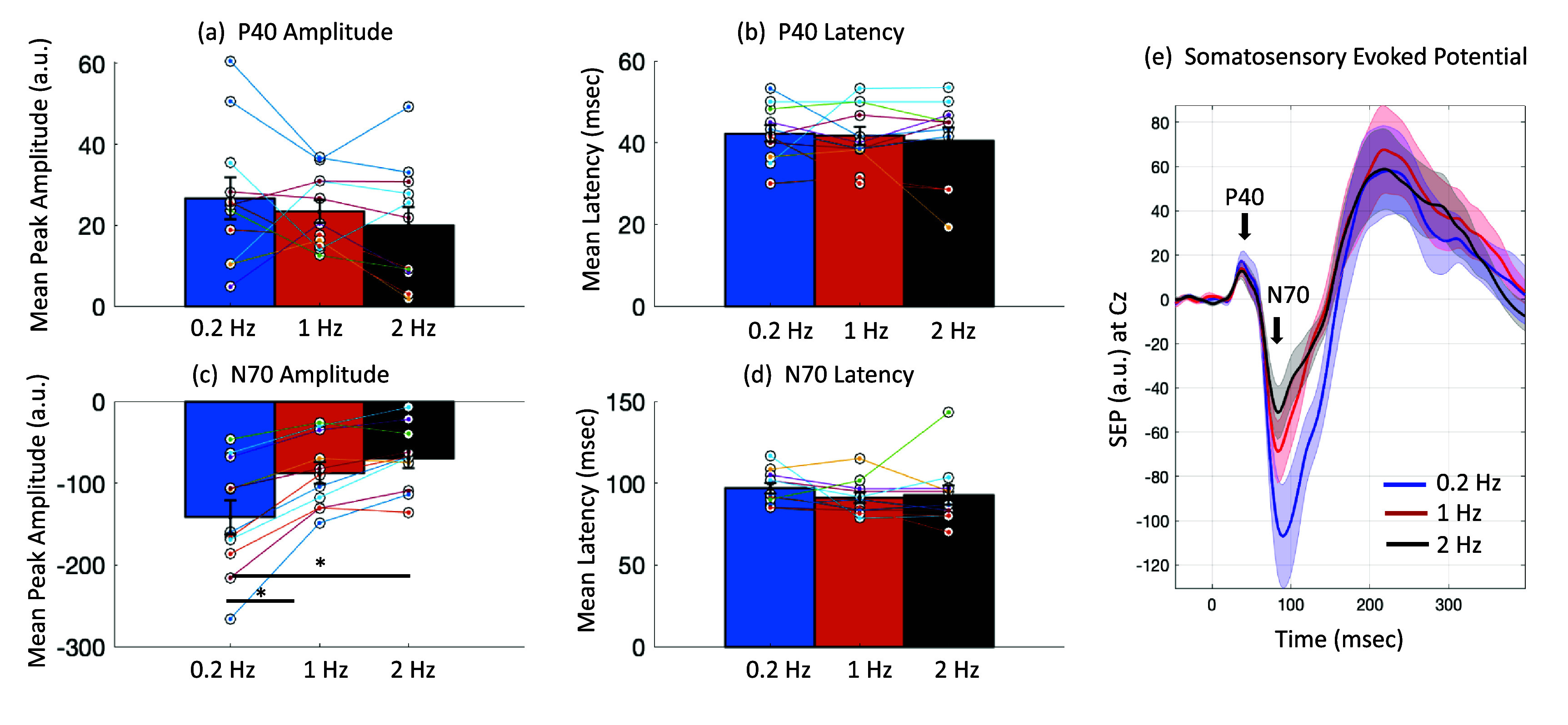
Effect of stimulation frequency on somatosensory evoked potential (SEP) components across participants: (a) P40 amplitude shows a small but not significant decrease with increasing stimulation frequency, (b) P40 latency appears stable, (c) N70 amplitude shows a significant decrease with increase in stimulation frequency (Friedman repeated measures test, *p* = 0.0004), (d) N70 latency does not show a significant change. (e) The mean SEPs across all participants for the three stimulation frequencies reflect these results. As frequency increases, P40 amplitude decreases slightly, N70 amplitude decreases substantially, and P40 and N70 latencies change little if at all.

*N70 attenuates with stimulation frequency:* We examined the P40 and N70 peaks at the three stimulation frequencies of 0.2 Hz, 1 Hz and 2 Hz (each with 10% ISI jitter). The spatial distributions of the *r*^2^s for these peaks were centered at the Cz electrode at all stimulation frequencies across participants (figure 3), as expected from the literature [39]. The average amplitudes and latency for P40 and N70 at Cz at the three stimulation frequencies are shown in table 1. The stimulation frequency had a minimal effect on the early P40 peak, but a marked effect on the N70 peak, which showed an average decrease of 39.4 ± 2.7% at 1 Hz and 57.2 ± 6.2% at 2 Hz, compared to that at 0.2 Hz. The results are shown in figure 4.

**Table 1.** Amplitude and latency for P40 and N70 at three stimulation frequencies and as a percent decrease from that at 0.2 Hz frequency (average ± standard error).

Stimulation frequency	P40 amplitude (a.u.)	N70 amplitude (a.u.)	P40 latency (ms)	N70 latency (ms)
0.2 Hz	26.7 ± 5.1	−141.5 ± 20.8	42.2 ± 2.1	96.8 ± 3.1
1 Hz	23.4 ± 2.7	−87.8 ± 13.1	41.6 ± 2.3	91.0 ± 3.2
2 Hz	20.1 ± 4.4	−69.9 ± 11.7	40.6 ± 3.2	92.4 ± 5.8

Stimulation frequency	P40 amplitude % Decrease	N70 amplitude % Decrease	P40 latency % Decrease	N70 latency % Decrease

1 Hz	6.2% ± 36.6	39.4% ± 2.7	0.0 ± 6.7	6.4% ± 3.5
2 Hz	27.8% ± 22.4	57.2% ± 6.2	4.0 ± 8.0	6.4% ± 6.9

Friedman analysis of variance for repeated measures was applied to the SEP N70 peak amplitude, with stimulation frequency as a factor. This indicated that a change in stimulation frequency significantly affected the SEP N70 peak amplitude (${\chi ^2}$ = 17.64; *p* = 0.0004, *w*= 0.80). Post hoc testing with 1-way ANOVA, revealed that the SEP N70 amplitude elicited by 0.2 Hz stimulation was significantly higher than that elicited by 1 Hz stimulation (*p* = 0.008) and 2 Hz stimulation (*p* = 0.0002). Friedman analysis of variance on the P40 peak amplitude did not show a significant effect of stimulation frequency (${\chi ^2}$= 5.09; *p* = 0.12, *w* = 0.23). It also did not show a significant effect of stimulation frequency on N70 latency (${\chi ^2}$= 5.14; *p* = 0.12, *w* = 0.23), nor P40 latency (${\chi ^2}$=0.2; *p* = 0.90, *w* = 0.01).

The effect of number of trials was assessed using *r*^2^, as a measure of SNR, for P40 and N70 amplitudes for 10 through 50 trials, in steps of 10. Neither P40 nor N70 showed a significant change (Friedman test, *p* > 0.05) in a smaller subset of trials.

The H-reflex-based selection criteria led to small variations in stimulation intensity across runs. The amount of intensity variation between runs was 3.53 ± 1.5% for 0.2 Hz to 1 Hz and 4.9 ± 1.9% for 0.2 Hz to 2 Hz. This variation was not statistically significant (${\chi ^2}$= 1.64, *p* = 0.44).

**Figure 5.**
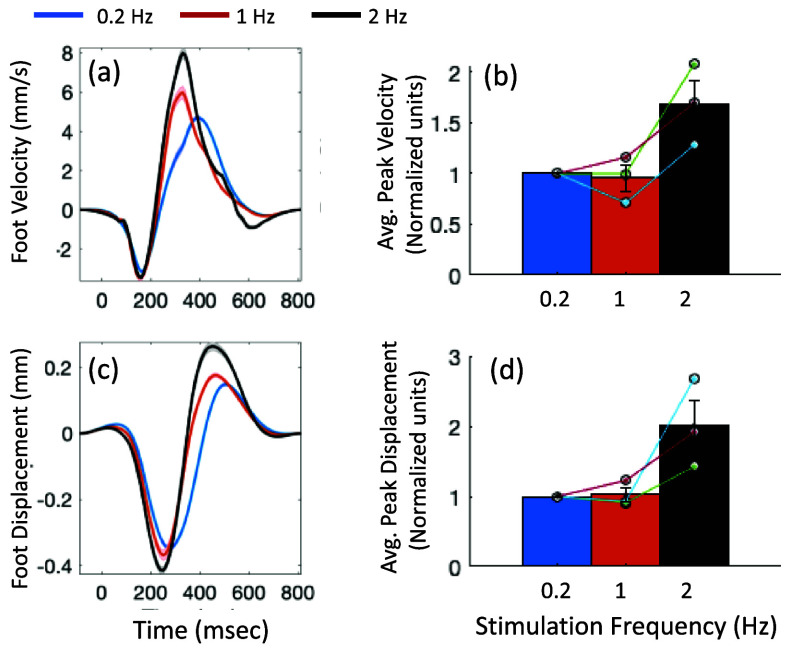
Add-on preliminary assessment of foot movement at the three stimulation frequencies, recorded in three participants. (a) and (c) Representative participant data shows the mean ± standard error of foot velocity and displacement across trials at the three stimulation frequencies. Time zero indicates stimulation onset. (b) and (d) Results across the three participants: mean and standard error of the peak velocity and displacement (normalized relative to their values at 0.2 Hz).

*Foot movement and stimulation frequency:* As an add-on preliminary assessment, we measured the foot movement and velocity in three participants, using an IMU placed on the participant’s foot. We observed an increase in foot displacement with increasing stimulation frequency from 0.2 Hz to 2 Hz (shown in figure 5). The mean and standard error of displacement and velocity across the epochs are shown for one of the participants in figures 5(a) and (c). The mean and standard error of velocity and displacement with respect to that at 0.2 Hz, across the three participants, were 0.94 ± 0.1 and 1.6 ± 0.2 at 1 and 2 Hz respectively (figure 5(b), and 1.0 ± 0.1 and 2.0 ± 0.4 at 1 and 2 Hz respectively (figure 5(d)). The Friedman Repeated Measures Tests for the velocity (${\chi ^2}$= 4.67, *p* = 0.097, *w* = 0.78) and displacement (${\chi ^2}$= 4.67, *p* = 0.097, *w* = 0.78) across the three stimulation frequencies, did not reach significance at *α* = 0.05, likely due to the small available *N*. Further testing of twitch induced movement changes at these low frequencies (0.2 Hz to 2 Hz) is required.

**Figure 6.**
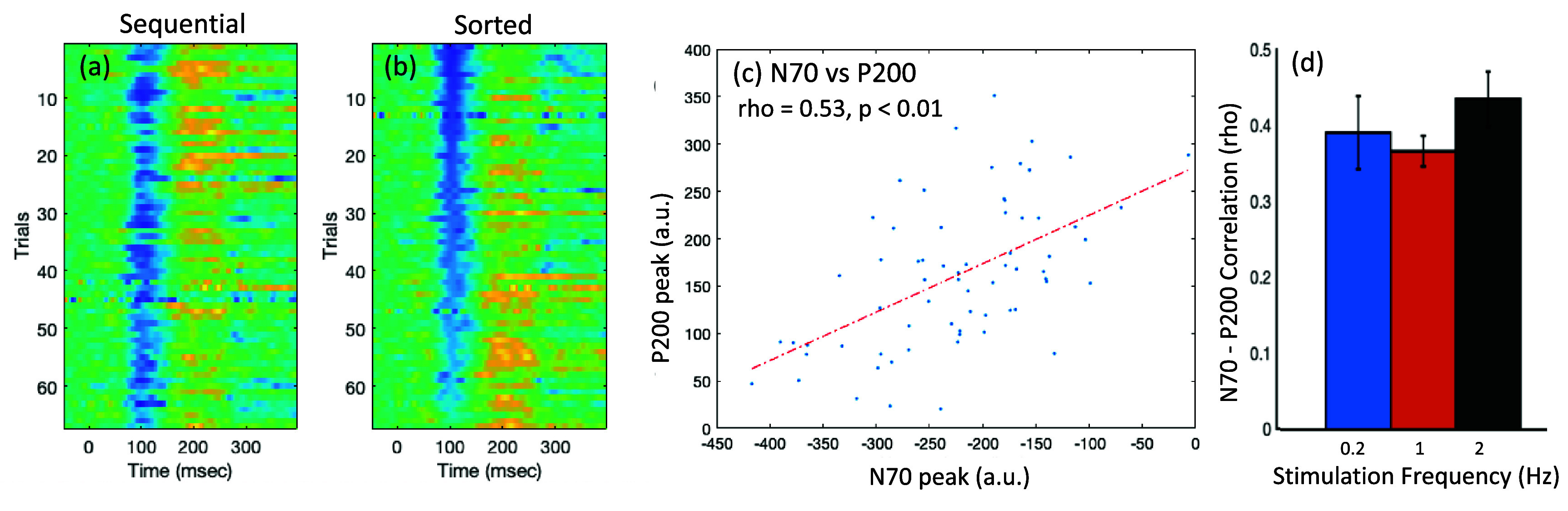
N70 and P200 association: (a)–(c) representative example from one participant: (a) trials shown in the order in which they were acquired. (b) Trials sorted by the N70 peak, show a reduction in N70 with a corresponding increase in the P200 peak, and (c) a significant correlation between N70 and P200 amplitudes across trials (*p* < 0.01). (d) Correlation between N70 and P200 amplitudes across participants, at the three stimulation frequencies.

*SEP N70 is associated with P200:* We also noted an association between the mid-latency N70 and longer-latency P200 cortical responses. For each participant and run, the N70 amplitude typically varied across trials within the expected range. However, in the trials in which the N70 peak was smaller in amplitude, the corresponding P200 appeared to be relatively higher in amplitude. Figures 6(a)–(c) illustrates this using a representative example. The panel (a) shows the trials in the order in which they were acquired, displaying a mixture of the N70 peak amplitudes across trials. The panel (b) shows the trials sorted by N70 deflections, displaying the corresponding gradation of the P200 peaks. Panel (c) shows the correlation between N70 and P200 for this individual. Correlation analysis across all participants showed that the N70 amplitude was consistently correlated with the P200 peak at all stimulation frequencies, with an average rho of 0.40 ± 0.05, 0.40 ± 0.02 and 0.43 ± 0.04 (figure 6(d)).

**Figure 7.**
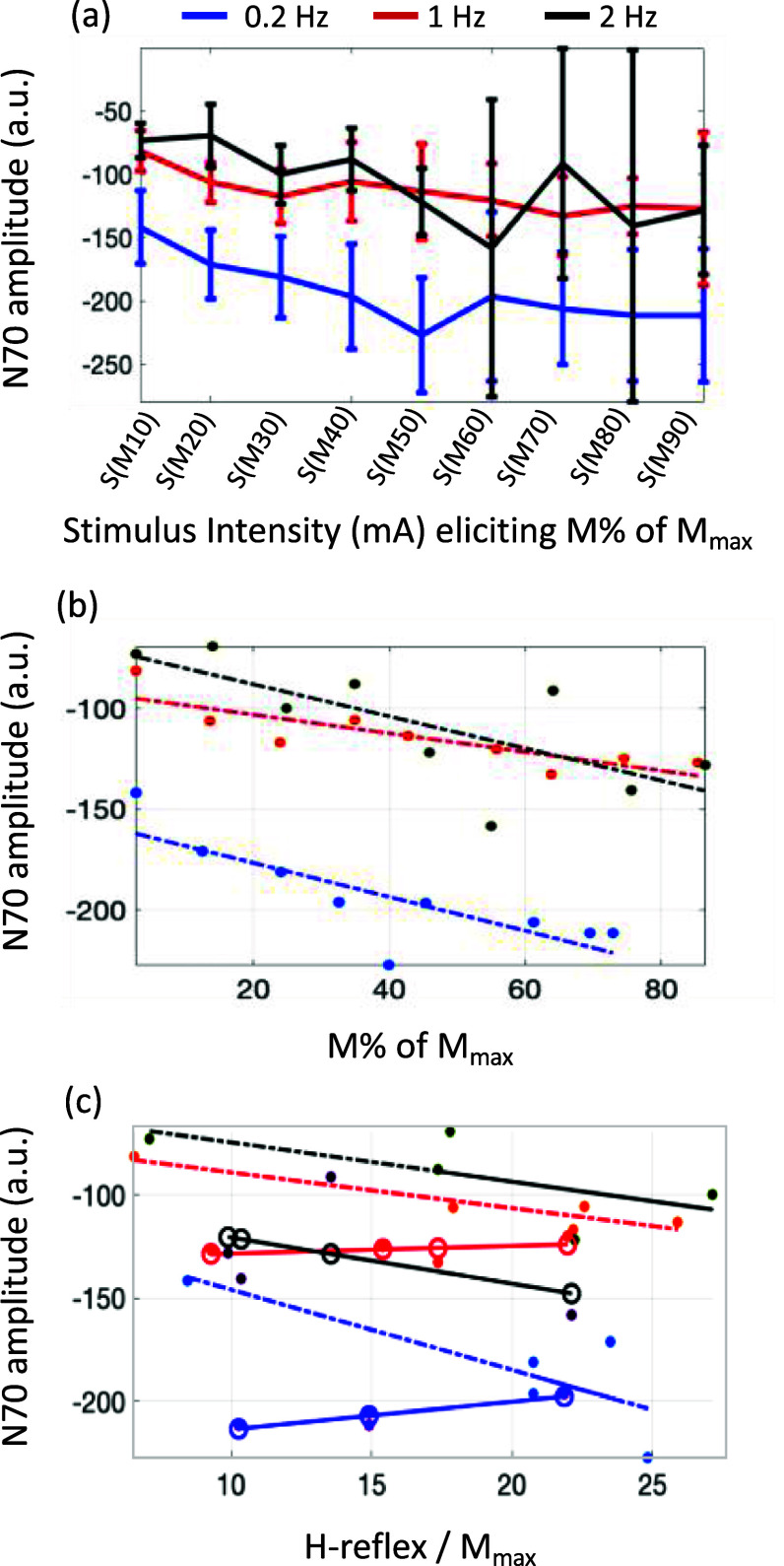
Effect of stimulus intensity on somatosensory evoked potential N70 amplitude, at three stimulation frequencies. (a) Mean and standard errors for N70 amplitude are shown at discretized and normalized stimulation intensities that elicit a percentage of *M*_max_. (b) Correlation between N70 amplitude and normalized M-wave was significant (*p* < 0.05) at all three stimulation frequencies (c) Correlation between N70 amplitude and *H*/*M*_max_ ratio was not significant (*p* > 0.05) at any stimulation frequency. The dots show the rising edge and the circles show the falling edge.

*SEP N70 amplitude is affected by stimulation intensity:* As EEG was also gathered during the recruitment curve data collection, we analyzed the effect of stimulation intensity on SEP N70, as it showed a high SNR even with a smaller subset of trials. This analysis was performed at discretized and normalized intensities. It showed a steady increase in the N70 peak with an increase in stimulation intensity (figure 7(a)) and was generally less variable at smaller stimulation intensities (<50% *M*_max_) than at higher intensities. This was observed at all the stimulation frequencies. The analysis of covariance showed that the N70 amplitude significantly differed with stimulation intensity (${\chi ^2}$= 28.3, *p* = 2.8 × 10^−5^) and stimulation frequency (${\chi ^2}$= 70.3, *p* = 5 × 10^−10^). Post hoc analysis of intercept estimates showed a significant difference between N70 amplitude at 0.2 Hz and 1 Hz (*p* = 0.0034), and between 0.2 Hz and 2 Hz (*p* = 0.0001), but not between 1 Hz and 2 Hz (*p* = 0.40). There was no significant interaction between stimulation frequency and stimulation intensity, (${\chi ^2}$= 0.66, *p* = 0.53), the N70 amplitude varied with intensity in a similar way across the three stimulation frequencies. Post hoc analyses of the slope estimates were not significant (*p* > 0.05).

*SEP N70 amplitude is correlated with M-wave:* Next, we assessed the relationship between the cortical response (N70 amplitude) and (a) the muscle activation (M-wave) and (b) the alpha-motoneuron activation (*H*/*M*_max_ ratio). The M-wave responses were normalized by the maximum M-wave response and discretized, as described in the data analysis section. A significant correlation was observed between N70 and M-wave (figure 7(b)) for all stimulation frequencies (0.2 Hz, rho = −0.83, *p* = 0.0043; 1 Hz, rho = −0.87, *p* = 0.0023; and 2 Hz, rho = −0.75, *p* = 0.013). The correlation between N70 and *H*/*M*_max_ ratio was weak and non-significant (*p* > 0.05), at all stimulation frequencies (rho = −0.15, 0.22, −0.13 for 0.2 Hz, 1 Hz and 2 Hz respectively). However, when the rising edge of the H-reflex was separated from the falling edge, the N70 showed more prominent trend of increasing with H-reflex during the rising edge and decreasing during the falling edge (figure 7(c)) for 0.2 Hz and 1 Hz.

## 5. Discussion

Many studies have separately measured SEPs [21, 40, 49, 50] and peripheral responses [2, 51] evoked by peripheral stimulation, but only few studies have measured cortical and peripheral responses concurrently [27]. We attempted to characterize the SEP obtained during the HrTNP protocol and examine the influence of some of the stimulation parameters, especially those that differ from the values recommended for SEP measurement.

*N70 decreases with stimulation frequency*

The N70-a mid-latency somatosensory response [30, 31]—was measured at the central scalp region [30] and its amplitude was observed to decrease significantly with increasing stimulation frequency, while its latency remained unchanged. N70 is considered to have a cortical source generator as it is abolished in cortical lesions [29]. Its attenuation at higher stimulation rates has been demonstrated in previous studies and is attributed to the ‘gating effect’ [52].

The gating effect has been described as the attenuation of the *direct* afferent response at the thalamus due to interference from the *secondary* afferent responses evoked by the muscle afferents in the stimulation of a mixed nerve. The direct afferent response is referred to as the orthodromic volley that travels via ascending sensory fibers to the cerebral cortex (evoking an SEP) and also travels largely monosynaptically to alpha-motoneurons in the spinal cord (eliciting the H-reflex). The secondary volley is generated by muscle afferents due to action potentials in the motor fibers that propagate as a descending efferent volley to the innervated muscle (at the motor threshold, which is a combination of M-wave and H-reflex). These action potentials lead to muscle contraction (twitch) and foot movement, with discharge from the joint and muscle proprioceptors (Golgi tendon organs) and slowly conducting cutaneous fibers [53]. This secondary muscle afferent response is transmitted to the cerebral cortex via rapidly conducting afferent fibers [54] associated with kinesthetic sensibility/proprioception and the transcortical proprioceptive reflex [55–57].

In addition to this peripherally driven gating or centripetal gating factor there is a cortically driven gating factor as well, called the centrifugal gating factor [58, 59]. It refers to the activation of inhibitory pathways by descending motor signals that gate interfering or task-irrelevant ascending afferent inputs [58] during pre-movement or active or passive movement [59–74].

The amount of gating has been shown to be influenced by parameters such as movement velocity, movement difficulty [75], and load type (proprioceptive position assessment rather than the force required to increase muscle torque) [75].

In the context of our study, as the participant had to maintain a constant level of voluntary contraction and an upright standing posture, both centrifugal and centripetal gating would be in play. With regard to centrifugal gating, at higher stimulation frequencies, when twitch-related foot movement becomes fast or more predictable, the muscle afferent response may become less task-relevant and potentially suppressed more by the motor cortex. With regard to centripetal gating, at higher stimulation frequencies, there would be a larger proprioceptor discharge due to the increase in muscle tension [76, 77], caused by the increase in the firing rate of the already recruited motor units (as the M-wave and the *H*/*M* ratio are maintained, which maintains the number of motor units recruited [78–80], including the motoneuron excitability). This increased (interfering) muscle afferent response will be suppressed (i.e. gated) by the thalamus.

As a supplementary test, we assessed the foot movement velocity and displacement in two participants and found an increasing amplitude with increasing stimulation frequency. However, this finding must be confirmed in a larger cohort.

*P40 remains unaffected with increase in stimulation frequency*

The tibial SEP had a P40 peak at the vertex (Cz), which is in agreement with the literature [21, 39, 81]. The amplitude and latency did not show a significant change with stimulation frequency. The amplitude decreased slightly, but not significantly, as the frequency increased. This can be explained by the nature of the source generator. P40 is thought to be generated by a thalamocortical source [27, 29, 49, 82], because it remains unchanged in cortical lesions [29]. Its latency is mainly known to be affected by thalamic lesions [83] or neurological disorders that affect fiber conduction times (such as multiple sclerosis [84]), which may explain the lack of change in its latency with stimulation frequency. Its amplitude reaches a maximum at the motor threshold [49], and the lack of its attenuation suggests that the gating effect from secondary afferents, elicited by stimulation-induced movement, occurs at the cortical level (also suggested by others [52]).

*SEP attenuation by habituation*

An alternative or complementary explanation for SEP attenuation is habituation due to the decreased novelty of the stimuli [85–87]. This was demonstrated in a study by Wang *et al* [87], where pairs of stimuli were presented at various ISIs with and without jitter, and an effect of habituation was observed on SEPs at shorter and constant ISIs [87]. Longer latency responses (P2 SEPs) were affected, probably due to the higher-order cognitive processes involved. To mitigate habituation in our study, the ISI for all stimulation frequencies was reasonably jittered (i.e. ±20% ISI for each rate); however, the variability in ISI jitter is reduced at higher stimulation frequencies, which can make the stimuli more easily predictable. The minimum jitter required to overcome somatosensory habituation with current stimulation parameters is not clear; therefore, it is uncertain how much of the SEP attenuation can be ascribed to stimulation-related habituation. For early and mid-latency responses—P40 and N70—we did not find a systematic reduction of amplitude across trials or between the start and end of a run at any stimulation frequency.

*Effect of stimulation intensity*

At low intensities, there is a small spinal reflex mediated response (H-reflex), a small peripheral motor axon-mediated response (M-wave), and a relatively smaller muscle contraction as compared to that at *M*_max_. At these relatively lower intensities, the orthodromic volley to the cerebral cortex elicits an N70, which is gated at higher frequencies owing to centripetal and centrifugal factors. The increase in stimulation intensity at constant stimulation frequency, while the H-reflex < *H*_max_ or until the *H*/*M*_max_ ratio is <0.5, elicits an M-wave and twitch response that is still relatively small. More sensory axons are depolarized, transmitting an overall larger volley to the motoneurons and cerebral cortex. This explains the initial increase in the SEP N70 amplitude (figure 7(a)), as also shown by other researchers [49]. With a further increase in the stimulation intensity, the antidromic activation of the motor axons starts to collide with the descending volley, reducing the H-reflex elicited from the spinal arc. Simultaneously, orthodromic depolarization in the peripherally descending motor axons (M-wave) increases, recruiting a larger number of motor units in the muscle and inducing a larger muscle afferent response. This increased interference from the muscle afferent would also have an increased centripetal gating effect on N70, which would explain the slight reduction and plateauing of N70 beyond S(M50), as shown in figure 7(a). These transitions were more apparent at 0.2 Hz as compared to the higher stimulation frequencies of 1 and 2 Hz. The N70 at 0.2 Hz also becomes more variable and more similar to the response at higher frequencies. This may be due to multiple competing gating factors in the cortex and spinal motoneurons at higher frequencies and intensities.

*Measurement of Foot Displacement*

We included a preliminary IMU test data to illustrate an approach that could be easily added to the HrTNP and SEP setup to monitor and measure foot movement variation. Note that induced foot movements can vary across people, depending on factors such as age, height, weight, or fitness level. While our three pilot subjects showed reasonably consistent results, the IMU methodology might require further refinement to cope with variability across larger samples—for example, positioning the IMUs on the lower leg rather than on the foot to better isolate the soleus contribution to the movement, and/or optimizing the analysis to extract more-invariant features.

*Deviation from recommended parameters for SEP measurement in an HrTNP protocol*

Measurement of the SEP concurrently with H-reflex and M-wave responses in an HrTNP protocol means that the SEP is measured using stimulation parameters different from those typically recommended for SEPs. We discuss the advantages and disadvantages of some of these variations that may be useful during concurrent SEP and HrTNP study designs.

(a) Stimulus pulse width: The recommended pulse width for eliciting SEPs is shorter (0.1–0.2 ms) than that used in the HrTNP protocols (1 ms). However, when measuring the afferent cortical response to a mixed nerve (e.g. tibial nerve), a longer pulse width is desirable, as it preferentially stimulates sensory fibers [88–91] due to their lower rheobase and longer strength-duration time constant than motor axons [92, 93]. A longer pulse width is also useful in non-invasive stimulation of nerves situated deeper under the skin, as it penetrates deeper into the subcutaneous tissue [32, 94]. It also reduces potential artifacts and cutaneous responses due to discomfort during stimulation, as smaller current amplitudes are required to reach the motor threshold. This is because a longer pulse width increases the total charge delivered to the nerve, making it easier to depolarize (according to the strength-duration curves [16]). The disadvantages are that it produces stronger plantarflexion contractions and can introduce noise from the activation of nearby non-targeted fibers or tissues, as more axons are recruited within the expanded stimulation zone [17]. SEPs from a longer pulse stimulation can be noisier if evoked at or below the motor threshold, as the smaller current amplitudes may not adequately excite enough nerve fibers to evoke an SEP [16], necessitating suprathreshold stimulation.
(b) Stimulation intensity: the recommended stimulation intensity for SEP measurement is typically a twitch threshold or 2–3 times the sensory perceptual threshold, while the stimulation intensity used in HrTNP protocols is suprathreshold, eliciting an M-wave that is 10%–20% of *M*_max_. This ensured that the effective stimulus strength was stable across sessions. At the same time, the relatively high stimulation intensity will also generate a larger muscle contraction, which can also be accompanied by late EMG bursts, perturbing the background EMG. To assess this, it may be worthwhile to monitor the accompanying muscle contraction or foot movement across sessions.
(c) Stimulation frequency: the recommended stimulation frequency for SEP measurement is 2–5 Hz. Combining higher stimulation frequencies with greater intensity and longer pulse width than typically recommended, reduces SEP amplitude, likely due to gating and possibly a deeper and wider spread of stimulation. Moreover, stimulating rapidly at the suprathreshold stimulation intensity while maintaining voluntary contraction (e.g. by standing upright) may produce strong muscle contractions and foot movement, prevent maintenance of stable background EMG, and create movement artifacts in the EEG. The recovery time of mid- and long-latency SEP components may be as long as 600 ms [49], which would affect SEPs at stimulation frequencies >1 Hz. Owing to these multiple factors, a stimulation frequency of ⩽1 Hz is preferable.
(d) Number of trials: the recommended number of trials for measuring an SEP is typically 200–500 (at a small pulse width and stimulation intensity at or below the motor threshold). In the HrTNP protocol, the pulse width is longer, but the stimulation intensity is suprathreshold due to the HrTNP criteria, evoking SEPs with a reasonable SNR, even with a small number of trials. However, SEP quality can be affected by the EEG hardware, acquisition setup, impedance, sampling rate, and environmental noise, and a larger number of trials may be required to overcome some of these issues. In this study, a dry active headset with a 300 Hz sampling rate was found to be suitable for extracting SEPs with reasonable SNR within 75 trials.

*Recommendations*

To record the SEP as part of an HrTNP protocol, we recommend the following:

(1) Stimulation pulse width of 1 ms (to preferentially activate the sensory axons);
(2) Stimulation intensity that elicits an H-reflex of ∼75% of *H*_max_ and an M-wave 10%–20% of *M*_max,_ with control of background EMG at ∼5%–10% MVC (which typically produces a visible large muscle twitch for every trial);
(3) Stimulation frequency ⩽1 Hz (to reduce SEP attenuation and participant fatigue);
(4) A jitter in the ISI (∼10%–15% of the ISI) (to minimize habituation);
(5) At least 50–75 trials;
(6) An EEG headset that is quick to setup and has multiple channels (for adequate denoising) and a sampling rate of at least 300 Hz, is resistant to movement artifacts and electrical noise, and can receive and synchronize isolated digital triggers. A dry active wireless EEG headset with these features functioned well in this current study.
(7) Care should be taken to monitor the participant for fatigue and eye-closing, (which can inject visual alpha-band activity into the EEG data. Talking and head movements should be minimized to reduce neural and movement artifacts in EEG.

*Potential Further Improvements*

(1) A higher EEG sampling rate can better define the short-latency components of the SEP.
(2) Selection of stimulation intensity from a recruitment curve could be formalized with an algorithm that selects based on predefined *M* and *H* percentages of *M*_max_ [46].
(3) The potential impact of small postural changes, which can affect the H-reflex [95] and perhaps the SEP, could be assessed using a floor mat with pressure sensors. Foot movement in response to stimulation, may be useful to monitor using wireless inertial sensors.

## 6. Conclusion

This study evaluated the influence of stimulation frequency (2 Hz, 1 Hz, or 0.2 Hz) on cortical SEP evoked by tibial nerve stimulation in a typical HrTNP protocol. The amplitude or latency of the SEP P40 component did not change significantly across the three stimulation frequencies. The amplitude of the SEP N70 component decreased with increasing frequency (−39% at 1 Hz, −57% at 2 Hz); N70 latency did not change significantly. Across the three frequencies, the N70 amplitude increased with the stimulation intensity and correlated with the M-wave size. A set of 50–75 trials was adequate for measuring the P40 and N70 components when acquiring data with a dry active EEG headset. Modest jitter in ISI can reduce habituation. Participant fatigue, foot movements, and eye-closure should be monitored. Studying the relationship between SEP and concurrent EMG responses may illuminate the corticospinal interactions underlying H-reflex operant conditioning and enhance the efficacy and range of its therapeutic uses.

## Data Availability

The data cannot be made publicly available upon publication due to legal restrictions preventing unrestricted public distribution. The data that support the findings of this study are available upon reasonable request from the authors.

## CRediT

Disha Gupta: Conceptualization, methodology, hardware/software, data collection, data analysis, results interpretation, writing-original draft preparation.

Jodi Brangaccio: Recruitment, data collection.

Helia Mojtabavi: Data collection.

Jonathan Carp: Data analysis, writing-reviewing.

Jonathan Wolpaw: Resources (IRB), conceptualization, writing-reviewing.

N. Jeremy Hill: Conceptualization, hardware/software, results interpretation, writing-reviewing.
